# Youth tobacco use cessation: 2008 update

**DOI:** 10.1186/1617-9625-5-3

**Published:** 2009-01-30

**Authors:** Steve Sussman, Ping Sun

**Affiliations:** 1 Departments of Psychology, Institute for Health Promotion and Disease Prevention Research, University of Southern California, Alhambra, CA, USA; 2 Department of Preventive Medicine, Institute for Health Promotion and Disease Prevention Research, University of Southern California, Alhambra, CA, USA

## Abstract

In this paper, an empirical review of 64 teen tobacco use cessation studies is provided. Examined include program contents, delivery modalities, number of contacts, and expected quit rates. In addition, means of recruitment and retention of smokers in programming are discussed. Also, promising contemporary methods of teen smoking cessation are examined, including use of pharmacologic adjuncts, electronic technology, and cigarette price increases (and no smoking policy). Conclusions are made regarding implications for developing and implementing teen tobacco use cessation programs.

## Introduction

Tobacco use is the most prevalent lifestyle-related cause of death worldwide [1,2]. Investigation of the prevalence of a regular pattern of tobacco use is illuminating. Daily cigarette smoking prevalence in the United States increases from approximately 4% among 12 year olds, to 8% among 16 year olds, 12% among 18 year olds, 15% among 20 year olds, and levels off among 26 year olds at 22%, and then drops by 4% among older age groups [3]. A similar pattern of regular smoking develops among youth in other countries, with some variation in the age range and steepness in increase [4]. The relatively steep daily smoking inflexion curve evident during the teen years supports the assertion that teen tobacco use cessation programming is needed among the world's youth [5] (also see , accessed on 12-20-07). In addition, internationally approximately 60%–85% of young tobacco users are likely to have made at least one quit attempt and failed [6-12]. It appears that youth do want to quit tobacco use, and most appear unable to quit on willpower alone [13].

There have been 8 systematic reviews of the teen smoking cessation literature thus far. First, Sussman, Lichtman, Ritt, and Pallonen [14] evaluated 34 programs, 17 smoking cessation trials and 17 smoking prevention trials for their impact on cessation of cigarette smoking. Sussman [15] provided an enlarged review of 66 cessation trials (which included single-subject designs), and 17 studies of self-initiated quitting. McDonald et al. [16] provided a re-review of the Sussman [15] study. Garrison et al. [17] reviewed 6 studies of relatively rigorous designs. Backinger et al. [18] did a qualitative review of prevention and cessation programs.

Then, Sussman, Sun, & Dent [10] completed the first formal meta-analysis of 48 studies, which included both experimental and quasi-experimental designs. Shortly thereafter Grimshaw & Stanton [19] provided a 2006 Cochrane meta-analysis of 15 studies. These two studies considered the previous studies from the previous reviews. Both meta-analyses included the same types of studies (randomized control trials, cluster-randomized control trials, and controlled trials [non-randomized]). Also, in both studies pregnant females were excluded such that both males and female participants were represented. However, in the Sussman, Sun, & Dent [10] meta-analysis, the comparison group had to be a minimally active standard care or no-treatment control group. (The Moolchan et al., [20] 2005 study was included as an exception; it included an active cognitive-behavioral plus placebo nicotine adjunct control group. This study was removed from the present analysis since the present analysis assesses only effects beyond standard care or minimal programming, not incremental effects of a component.) In the Grimshaw & Stanton study, the comparison condition could be an "active" or "control" condition. On the other hand, only a quit point was needed for inclusion in the Sussman and colleagues meta-analysis (including end of program or follow-up), whereas a minimum of 6-months follow-up was a standard for inclusion in the other meta-analysis.

Both meta-analyses reached the same general conclusions, which were similar to the other reviews; that is, it appeared that some combination of motivation and cognitive behavioral strategies provided efficacious teen cessation results, whereas pharmacologic treatments did not, although much more research was needed. Finally, Gervais and colleagues [7] provided an empirical review of 16 randomized controlled trials (RCTs) derived from previous reviews and data searches up to November, 2006, and they provided the same general results.

In the present paper, a 9^th ^review is provided. Among the variables examined include the most effective tobacco use cessation program contents, modalities of delivery, number of contacts, and expected quit rates at follow-ups. In addition, means of recruitment and retention of smokers into programming, and suggestions on the lead time needed for a measurable effect, are discussed. In addition, some promising contemporary methods of teen smoking cessation are examined. These include use pharmacologic adjuncts, use of computers or other electronic technology, and use of cigarette price increases. The hope is that the information in this review will have implications for development, implementation and evaluation of teen cessation programming worldwide.

## Description of the 64 controlled studies in the present review

### Study selection

The general protocol of locating studies was through multiple sources. We compiled searches of PsycINFO, MedINFO, the Google web engine (using the terms "teen", "adolescent", "tobacco use", "smoking", cessation", "programs"), and word of mouth. The duration of that search was from January, 1970 through December 2007. Included was any article or report in the English language that included data regarding the contents of a teen smoking cessation effort, quit rates, and a through-study age range of 12 to 19 years old. Studies that included fewer than 8 cigarette smokers at baseline were excluded due to extremely small sample size (less than 5 smokers per condition). Tobacco related interventions for pregnant females were not included, so that all studies involved both genders as subjects. Data that might be available through surveys of practitioners in the field was not selected.

Finally, only studies that included a control condition were selected. A total of 130 studies were located; only 64 of these studies were controlled trials. These included 47 of the 48 controlled trials from Sussman, Sun, & Dent [10] (the Moochan study was deleted because it did not include a minimal program control condition), 3 studies from the Grimshaw & Stanton [19] review that were not contained in the concurrent review [21-23], and 14 additional studies located subsequently to both of these reviews [24-37]. The data used for the present analysis and abbreviated references for these 64 studies are contained in an additional text file [see Additional file 1].

### What has been completed outside of the U.S. and where

One limitation of a review of teen tobacco use cessation programs is that relatively few studies have been conducted compared to adult cessation programs [1], and even fewer teen tobacco use cessation studies have been conducted outside the United States. In the present study 64 controlled teen cessation programs are examined, and 17 studies were completed outside of the U.S. Of these studies, 4 were from Australia, 3 were from Canada, 1 was from China, 1 was from Finland, 2 were from New Zealand, 1 was from Singapore, 1 was from Switzerland, and 4 were from the UK. Among the 8 countries outside of the U.S. that conducted published teen cessation work in English, aside from the study in China, all were high per capita income countries. Results have failed to be found to vary systematically by whether or not data are from the U.S. or not [10,15].

### Adequacy of designs, data quality and collection for the present review

#### Research designs

The review discerned the difference between treatment group quit rates and naturally occurring or comparison group minimal treatment quit rates (e.g., standard care) [38,39]. One method of doing this is through use of randomized designs, in which groups are assigned to conditions (program versus control group) based on a "flip of a coin." This design best controls for confounders that might interfere with interpretation of results. The World Health Organization (WHO) refers to this level of evidence as being "probable;" that is, the expected changes were unlikely to have occurred by chance (32 RCTs were located). A second kind of design includes a control group that is not assigned randomly. This provides a crude means of gauging what the quit rate might have been if the program group had not received the treatment. WHO refers to this level of evidence as "plausible;" the expected changes that occurred were greater than could be explained by any other external influences (31 quasi-experimental trials were located). We also included multiple-baseline designs, or other means of finding a control group quit rate (e.g., data that aggregates quit rates from other studies in the region). WHO describes this level of evidence as "adequate" (only one multiple baseline trial was located). We failed to find variation in results as a function of level of evidence both in the Sussman, Sun, & Dent [10] review and with inclusion of 17 additional controlled studies in the present review. Thus, we do not divide the presentation of results by level of evidence. The overall level of evidence for the studies in this review is within a range from plausible to probable. Studies in general would be considered efficacy studies, being conducted under ideal conditions. However, most of the studies confronted real world settings, and might be considered effectiveness trials as such.

As mentioned previously, these 64 controlled trials were derived from 130 total studies; 50% of the total pool of studies uncovered lacked control conditions, and could not be included in the review. Approximately 1/3 of the studies post 2000 were single-group designs, suggesting some improvement in designs of these types of studies since that time. Also, approximately 1/3 of teen cessation studies were published in 2000 or later (n = 42). Thus, interest in teen cessation appears to be increasing.

While validity scores were not used in the present study or in Sussman, Sun, & Dent [10] approximately 66% of the studies in the current review would be considered of moderate or high quality using the McDonald et al. [16] criteria (theoretical fidelity, implementation compliance, research design, sample size, and post-treatment follow-up; ratings of 2 = high, 1 = low, and 0 = no report for each criterion which were summed to create a validity score). The conclusions of the McDonald et al. study were the same as in the Sussman, Sun, & Dent [10] study.

#### Data quality and collection

Only 27 of 64 studies (42%) described ethnicity of subject in the present review. The prevalence of missing data on ethnicity was too high to include this variable in any analyses. Collection of ethnicity data should become a regular process in these studies, and programming needs to be offered and evaluated in areas with higher racial minority concentrations to assess generalizability of programming to different ethnic groups.

Also, insufficient data was available to calculate reduction in average level or duration of baseline cigarette smoking among those who did and did not quit cigarette smoking after receiving programming, particularly at follow-up time points. Better measurement of current smoking behavior is needed in these studies.

Average rate of reach to target recruitment was approximately 50%, and average retention rate was approximately 75% for follow-up. Though there was large variation on these measures, it would appear that the data collection was adequate considered across all studies.

### Adequacy of program implementation

It would appear that program implementation is one of the relatively strong features of published programs. Oftentimes, programs were somewhat eclectic in contents, but research teams reported that they implemented these contents fairly well. There was little evidence provided of disruption during implementation, omitted sessions, or reinvention. On the other hand, specific documentation of fidelity of implementation was not provided in most studies. Certainly, major changes have occurred in program implementation. For example, NOT, which was developed to be implemented to same sex groups, generally is implemented to both genders in field studies, with little apparent negative impact on results (Dino and Horn, personal communication by program developers, November, 2007). Most studies don't inform the reader on provider characteristics (e.g., training, age, and experience). None discuss issues related to cost and feasibility of implementation. Thus, the program implementation fidelity "transparency" is not clear. Research is needed in this domain of inquiry.

### Recruitment strategies

Across the 64 studies, direct interpersonal contact of treatment agent with potential participants and recruitment in contexts that include most of its members as potential participants (e.g., classrooms) led to relatively high reach (over 35%). The most popular recruitment strategies were:

• word of mouth (n = 24 studies)

• public announcements (n = 17)

• screening (n = 17)

• money, movie tickets, gift certificates (n = 14)

• class release time (n = 12)

• use of posters (n = 12)

• media campaigns/newspaper ads (n = 9)

• policies such as mandatory attendance (n = 8)

• referrals (n = 7)

• flyers (n = 6)

• part of a classroom program (n = 6)

• presentation to a group (n = 5)

• gatekeepers' support (n = 5)

• use of class credit (n = 4)

• use of contests (n = 3)

• use of a display table (n = 2)

• social influence (n = 2)

• peer supporters to recruit (n = 2)

• use of community or school events (n = 1)

It is not possible to discern which recruitment methods are the most effective from this pool of studies. It would appear that use of multiple strategies work better than use of one strategy, except in situations in which youth are "captive audiences" (e.g., classroom program or mandatory attendance in a program), where recruitment is not a major issue.

### Representativeness of the data

Youngest to oldest age at baseline to last follow-up was an average of 14 and 19 years. Baseline smoking averaged approximately 10 cigarettes per day (cpd). The average sample size was a mean of 414 (range 12 to 3800; sd = 588). In addition, an average of 51% of subjects was female. In the Sussman, Sun, & Dent [10] review, in preliminary analyses we failed to find bias in effect size as a function of variability in sample size, year of publication of study, due to random assignment or not, follow-up retention, average level of baseline smoking (though various studies do find lower quit rates among heavier smokers [39]), country of study (U.S. or Other), gender, ethnicity, mean age, age range, program reach, and years data were collected. The addition of 17 studies does not alter these results. It would appear that this data is representative of subjects involved in these types of studies.

### Variables examined in the analysis

As in Sussman, Sun, & Dent [10], we examined the mean estimates for four main predictors of outcomes.

• Five types of contents: (1) social influence, (2) cognitive-behavioral, (3) motivation, (4) medical, and (5) other (i.e., supply reduction and affect clarification)

• Seven modalities: (1) classroom, (2) school-clinics, (3) medical clinics, (4) family, (5) system-wide, (6) computer, (7) other settings (sensory deprivation, court diversion, worksite, shopping mall/home, and dormitory).

• #Sessions: 1–4, 5–8, 9+ (3 categories)

• Length of follow-up: 0–3, 4–12, and > 12 months past immediate posttest (3 categories)

We merged sensory deprivation and court diversion from the previous study (they were treated as separate modalities with one study each in the 2006 study), and we labeled the category "other settings." In addition, we added three new studies to this category. These were: dormitory [21], shopping mall and home [22], and worksite [34].

Smoking data was converted into cigarettes per day equivalents, and was determined through self-reports. Biochemical methods were applied in 38 (59%) of the studies. A standard definition of baseline smoking and quitting is not used across studies. In some studies quitting is measured as no smoking over at least one-week duration, whereas in other studies quitting is measured as no smoking over the last 30 days. Variation in measures introduces error variance, which could make it more difficult to find an effect.

### Adequacy of data analysis

Most teen cessation studies were under-powered statistically; that is, the sample sizes tended to be too small to detect differences between program and control means with reasonable certainty [38,40]. Also, most studies failed to use clustered analyses as they were not yet popularly used with clustered data. Otherwise, previous studies provided data analysis techniques in general that were appropriate for the years in which they were conducted.

The primary endpoint in the present review was percent quit-rate (P). The data entered were intent-to-treat percent quit data. The net effect definition in this study was a risk difference (RD); also called "absolute risk reduction." The RD here was percentage of subjects that quit tobacco in a program condition minus the percentage of subjects that quit tobacco in a control condition.

Use of Intent-To-Treat (ITT) analysis was one way to deal with attrition from studies. That is, subjects who dropped out of the studies were considered to not have quit smoking. However, ITT analysis is not without its drawbacks when comparing conditions. ITT analysis could bias the results if there was differential attrition by condition. However, we failed to find differential attrition by condition averaged over the studies, so that ITT was used correctly herein.

In the Sussman, Sun, & Dent meta-analysis, data were entered as intent-to-treat (ITT) quit rates (not compliance sample rates), and weighted least squares random effects models were used to pool results from study net effect estimates. When pooling studies, they were weighted by sample size, and were adjusted for follow-up duration (1–3 month, 3–12 month, or greater than 12 months). Analyses were supplemented with use of forest plots. The same analyses were repeated in the present study.

## Results of current review

### Overall effect

The program mean estimate was 11.79 (error = 1.10, t value = 10.72, p < .001, two-tailed), and the control mean estimate was 7.53 (error = 1.11, t value = 6.85, p < .001, two-tailed). The overall absolute risk reduction effect was a program advantage of absolute effect = 4.26% (relative increase for treatment: (11.79–7.53)/7.53 = 57% reduction). Thus, after a 27% increase in number of studies in the pool, the overall outcomes are slightly higher than the previous meta-analysis (2.90% advantage with n = 48), though the effect size is still not large.

### Theoretical frameworks

"Theory" refers to the theoretical content of the program. "Modality" refers to the community unit within which the cessation program is implemented. Collapsed across different modalities of programming, a total of five types of theoretical foci were reflected in these studies [10]. These categories were: (1) social influence, (2) cognitive-behavioral, (3) motivation, (4) medical, and (5) other (i.e., supply reduction and affect clarification). The purpose of social influence-oriented programming was to combat social influences that serve to promote or maintain smoking. Such information included refusal assertion skill instruction, instruction in awareness of tobacco industry promotions, media and peer social influences, and correction of social informational inaccuracies. This also includes advocacy (activism) techniques to advocate cessation by empowering teens to protest against the tobacco industry or otherwise act directly on their social environment to induce change (e.g., trying to reduce exposure to passive smoking).

Instruction of cognitive-behavioral coping techniques focus on uncovering the topography of ones tobacco use (e.g., reasons for smoking and quitting, self-monitoring) and how to cope effectively with stress (e.g., seek out social support, relaxation, wait out urges, self-management, problem solving). In addition, decision making is a key aspect of this approach, to help consider choices, select a choice, and choose which behaviors to follow through on.

Motivation enhancement techniques serve to clarify desire for change and reduce ambivalence toward change. This may include, but is not restricted to, a specific strategy such as motivational interviewing. Motivation enhancement helps participants to clarify their direction of change and increases their willingness to change. Motivation enhancement may also include use of response-contingent reinforcement, which reinforces quit-behavior with the chance for extrinsic rewards such as money or prizes. Motivation-enhancement also may include stages-of-change techniques. In Transtheoretical (stages of change) model-based work, program material is framed for the participants' stage of change, to help motivate subjects to move through the quitting process. These programs often tend to be combined with cognitive-behavioral material. However, a main emphasis of the program is its inclusion of motivation enhancement.

The medical category refers to use of means to ease physical effects of withdrawal (use of pharmacological adjuncts or substitutes), and/or emphasis on recovery from addiction (e.g., 12-steps), or use of alternative medicine techniques such as acupuncture.

An "Other" category includes categories that didn't fit elsewhere; supply reduction and affect clarification approaches. Supply reduction involves arranging the physical environment such that tobacco is more difficult to obtain or use (e.g., price increases or restricted access). Affect clarification involves techniques to clarify and remove conflicted affect, and thereby permit pursuit of health including cigarette smoking cessation.

The cessation theory outcomes for the 64 studies revealed the same pattern as the 2006 analysis [10], as shown in Table 1. Effects were notable for social influences, cognitive-behavioral, and motivation enhancement programming. Results also appeared for medical programming (error = 3.22, t = 4.93, p < .001), in the current analysis since the number of studies increased from 1 to 3. However, the number of studies is too small to infer consistent effects. Significance values from the previous study on the three outcomes mentioned is maintained from the previous meta-analysis to this one, and one new effect appeared albeit based on very few studies.

**Table 1:** Treatment Means: 2006 and Current Analysis Stratified by Theory

Theory	2006 Estimate	Current Estimate
Social influence (8, 11)	3.77	4.34
Cognitive-behavior (17, 22)	4.72	5.32
Motivation (15, 22)	3.66	3.97
Medical (1, 3)	13.16	15.86
Other (6, 6)	-0.16	-0.17

Note. The information in parentheses indicates the number of studies in 2006 and then the current analysis. Moolchan et al. (2005) had used a medical model but this study is not included in this table's current estimate because it had not included a standard care control condition.

### Modality of programming

There were 7 groupings of community units within which the cessation program was examined. The significant effects of classroom and school-based clinic modalities revealed in the previous meta-analysis were maintained with the larger sample, as depicted in Table 2. Medical settings now showed a significant effect (error = 1.88, t = 2.46, p < .05). Other effects still were not significant though computer-based programming still looks promising. Too few studies were in family (n = 1) or other settings (n = 3) modalities to make confident inferences.

**Table 2:** Treatment Means: 2006 and Current Analysis Stratified by Modality

Modality	2006 Estimate	Current Estimate
Classroom (7, 11)	4.15	4.21
School Clinics (25, 29)	5.62	6.30
Medical Clinics (5, 9)	2.40	4.62
Family (1, 1)	21.37	19.10
System-Wide (5, 6)	-0.22	0.81
Computer (2, 3)	5.60	5.40
Other Settings (2, 5)	1.45	3.92

Note. The information in parentheses indicates the number of studies in 2006 and then the current analysis, 2007. Moolchan et al. (2005) had used a medical modality but this study is not included in the current estimate because it had not included a standard care control condition.

One limitation in trying to differentiate theory from modality is that these are not orthogonal categorizations. In the current sample, 7 of 11 classroom-based studies involve social-influence manipulations, 20 of 29 school-clinic studies are cognitive-behavioral, 8 of 9 medical clinic studies are motivation-enhancement-based, 4 of 6 system-wide studies are in the "other" theory category, and 2 of 3 computer-based studies are motivation-enhancement based.

### Number of sessions and length of follow-up

Relatively higher quit rates were found for programs having at least 5 sessions (> 4 sessions, 6% increase compared to controls). This pattern of results was maintained as in the 2006 meta-analysis, as shown in Table 3.

**Table 3:** Treatment Means: 2006 and Current Analysis Stratified by Number of Sessions

Number of sessions	2006 Estimate	Current Estimate
1–4 (17, 26)	-0.08	3.20
5–8 (15, 20)	6.43	6.24
9+ (15, 18)	4.47	4.20

Note. The information in parentheses indicates the number of studies in 2006 and then the current analysis, 2007. Moolchan et al. (2005) had 13 sessions but this study is not included in this table's current estimate because it had not included a standard care control condition.

Effects were maintained at short term (1 year or less) and longer-term (> than 1 year) follow-ups in both the 2006 and present analysis, as shown in Table 4. The number of studies examining greater than 12 month follow-ups increased from 5 to 8 in the present analysis, and yet the effect still held up well. More studies with longer-term follow-ups are needed, though these data remain promising, and suggest that across studies most teen cessation rates tend not to decrease much over time. Programs targeting teen smoking cessation do appear to be efficacious.

**Table 4:** Treatment Means: 2006 and Current Analysis Stratified by Follow-up Duration

Follow-up Duration	2006 Estimate	Current Estimate
0–3 month (36, 38)	3.88	4.17
4–12 month (21, 29)	2.92	4.06
> 12 month (5, 8)	6.62	6.78

Note. The information in parentheses indicates the number of studies in 2006 and then the current analysis. Moolchan et al. (2005) had a 3 month follow-up but this study is not included in this table's current estimate because it had not included a standard care control condition.

## Examination of programs of particular contemporary interest

### Pharmacologic adjuncts

There is a strong interest in the promise of use of pharmacologic adjuncts for teens since these treatment agents have been very useful among adults [1]. This type of programming generally is constructed as an addition to other treatment programming such as cognitive-behavioral treatment. That is, most of these types of trials compare an active treatment to the active treatment plus a pharmacologic adjunct. Hence, these types of designs are not contained in the meta-analysis as the comparison condition is an "active" control.

A total of 10 studies have been completed thus far (Hanson et al. [41], Hurt et al. [42]; Killen et al. [43] [both nicotine patch and Buproprion were examined]; Moolchan et al. [20] [both nicotine gum and patch were examined]; Muramoto et al. [44]; Niederhofer & Huber [45]; Roddy et al. [46]; Smith et al. [47]; Sussman et al. [48]; Upadhyaya and colleagues [49]). Seven of these studies utilized comparison groups, which were cognitive-behavioral, standard cessation counseling (including instruction of coping skills). Unfortunately, the use of a pharmacologic adjunct failed to show an incremental effect among teens in 5 of the 7 comparison group trials. The mean incremental effect at last follow-up for nicotine gum was 2.5% (2 controlled studies; 4% and 1%), for nicotine patch was 4.5% (4 controlled studies; 2%, 15%, 1%, and 0%), and for buproprion was 13% (3 controlled studies; 1%, 1%, and 37%). Only Moolchan and colleagues found a treatment effect for nicotine gum (4%) and the nicotine patch (15%; 6-month trial, n = 120). In addition, only Niederhofer and colleagues found an effect for buproprion (37%; 3-month trial, n = 22). While effects were not promising, one study found a reduction in withdrawal symptoms [42].

Three of the 10 studies were single-group in design [42,47,49]; the Hurt [42] and Smith [47] studies each revealed a 5% quit rate, examining use of the nicotine patch; Upadhyaya and colleagues [49] revealed a 31% quit rate after 4 weeks of taking Buproprion SR [n = 16]). While difficult to interpret, it is not likely that an effect was achieved in 2 of the 3 single group studies, considering naturally occurring quit rates among teens.

Even if medication was helpful with teens, Shelley et al. [50] found that in the U.S. in 2000 only 16% of physicians and 12% of dentists advised their teen smoker patients to quit smoking. Thus, major changes in medical settings would be needed in dissemination work. Recently, Pfizer is testing the efficacy of varenicline, a partial nicotinergic receptor agonist, with teens (; accessed 12-18-07). This drug has been found to be highly effective with adults, but it will take several years to learn whether or not it is of assistance with teens.

### Effects of internet/txt messaging

Another area of current interest is in the use of electronic communications technology to assist in teen cessation. A total of 6 studies were located (Chen & Yeh [51]; Mermelstein & Turner [52]; Patten et al. [53]; Rabias et al. [54]; Rodgers et al. [33]; Tossman & Lang [55]). Only two of these studies (Rabias et al. [54]; Rodgers et al. [33]) were included in the 64-study review. Chen & Yeh [51] compared a smoking cessation group plus internet-assisted program instruction versus a standard care comparison group in a 6-week pretest-posttest quasi-experimental design consisting of a total of 77 senior high school teens in Taiwan. The program condition resulted in a higher reduction in rates of daily smoking (21% reduction versus a 2.5% increase) and a greater number of quit attempts relative to the control group (an average of 1 more quit attempt during the 6-week period). Youth appeared favorable to including the internet component. However, quit data, or means to estimate it, was not provided in the paper.

Mermelstein & Turner [52] found at 29 high schools (n = 351) among 14–19 year olds in a clustered-randomized control trial that use of a school-based clinic teen tobacco use cessation program (NOT) plus use of a website and proactive phone calls produced marginally higher quit rates at a 3-month follow-up than use of the clinic alone (14% vs. 7%; 7-day quit rate).

Patten et al. [53] contrasted a 4-session office clinic-based program (n = 139) involving motivational interviewing and problem solving among 11–18 year olds against a home-based internet program (Stamp Out Smokes; access was provided for 24 weeks; 66% stopped using the internet program by the 3rd week of the program) in a randomized control trial. The 30-day ITT quit rates at 36 weeks favored the office based program, 13% vs. 6%, respectively, though the outcomes failed to reach statistical significance. Tossman and Lang [55] in Germany with a single group design found 48% quit rates among 16–18 year olds (7-day) obtained one month after quit day with use of an individual-tailored internet program "rauchfrei" ("smoke-free").

Rabius et al. [54] contrasted 5-session telephone counseling vs. self-help booklets in a randomized controlled trial among 18–25 year olds (12% of the sample of 420 youth was still teens). At 6-month follow-up 10% vs. 3% had quit (ITT quit rate, last 48 hours). Finally, Rodgers et al. [33] provided a randomized controlled trial. This study included 617 teen smokers, who received personalized cognitive-behavioral oriented cell phone text messaging for 1 week before and 4 weeks after quit day versus a control group (bi-monthly general text messages to keep them involved in the study). While earlier results looked promising (14% vs. 6% ITT at 6-weeks, 29% vs. 19% at 12 weeks), results at 6-month follow-up were not (25% vs. 24%). It appears that use of telephone counseling is promising. Use of the internet or text messaging also may be promising if programming is bolstered over a long period. Much more research is needed in this arena, as these different communication devices will begin to interface and become more interactive.

### Price of cigarettes, restrictions on smoking, and teen cessation

At present, we are aware of only one study that examines the relations between price of cigarettes and teen smoking cessation. Tauras & Chaloupka [56] completed this work, using the Monitoring the Future High School Senior data. These researchers reported that the price elasticity of male cessation ranges from 1.07 to 1.17 and has an average elasticity of 1.12. The price elasticity of female smoking cessation ranges from 1.17 to 1.21 and has an average elasticity of 1.19. These estimates imply that a 10% increase in the real price of cigarettes will increase the probability of smoking cessation by approximately 11% and 12% for 18 year old men and women respectively (3.5% among young adults [57]). Maybe for teens increasing prices significantly reduces prevalence 6–7% (Chaloupka, personal communication, March, 2007).

State-level policies restricting smoking in private workplaces have a positive impact on the probability of cessation among employed young adult females. Other restrictions on smoking in public places seem to have little impact on female smoking cessation decisions. In general, laws restricting smoking in private worksites and public places have no significant impact on young adult male smoking cessation decisions [56], though Tauras [57] did find an effect, albeit equivocal, on young adult cessation as a function of restrictions in private worksites and public settings other than restaurants (both genders; weaker effect among males). There are no data on teens at present, except for those summarized in the analysis of 64 studies which failed to find an effect.

Regarding limiting retail access to tobacco products, Chen & Forster [58] conducted a two-group experimental study, involving cross-sectional surveys of 8^th^, 9^th^, and 10^th ^graders from a total of 14 communities, that demonstrated an effect on reducing prevalence of daily smoking by mobilizing community members to limit retail access to youth. This effect was found up to a five-year follow-up; however, cessation of smoking or other tobacco use was not accessed.

## Conclusion

Based on the empirical review of 64 programs, and review of contemporary possibilities, we propose the following suggestions for advancing youth cessation internationally. First, cessation programming should be delivered in a context which is structured for youth, such as the school, sports club, or health clinic, because youth tend not to impose structured situations on themselves (e.g., by keeping an appointment book). Also, programming should consist of at least 5 sessions. Also, programming should be as fun as possible, involving games, dramatizations, and use of alternative medicine concepts. Youth will want to remain in a program that is interesting [4,15].

The contents of teen cessation programming should emphasize cognitive-behavioral, motivation theory-related, and some social influences contents. For example, awareness of the changes that gradually occur as a function of continued smoking (e.g., increased stress, decreased mood) and quitting (e.g., decreased stress, improved mood) need instruction, along with means to help motivate youth to overcome ambivalence toward quitting. Instruction on how to avoid or counteract cigarette smoking social influence situations should be instructed. Also, methods of quitting and how to cope with stressful situations more effectively should be instructed. It is not clear whether programming that combines all three types of programming would be superior, or whether different programming might be relatively effective with different youth (e.g., at different durations of lifetime smoking) but, for the time being, it might be best to combine elements of each. Tentatively, programming that monopolizes numerous channels of communication would appear best [59]. Thus, use of classroom based programs, or school-based clinics, supplemented with computer based modalities, parent groups, mass media messages, or any other modalities that could be supported within a community, would be of most promise. Program efficacy is positively related to dosage, up to 5 cessation sessions at which point there is no apparent incremental effect of including additional sessions.

In 2004, the U.S. DHHS published the first guide for making informed decisions regarding teen cigarette smoking cessation ([60]; ; accessed on December 20, 2007). The recommendations made are consistent with the findings of the present analysis, and provide the practitioner with information on how to select a cessation program, what contents to include, how to implement a program, and how to evaluate it. At present, there are only two Substance Abuse Mental Health Services Administration (SAMHSA) model teen cigarette smoking cessation programs, Projects NOT and EX. Both of these projects may be purchased and utilized, and information on them is available on the SAMSHA model programs web site . Interestingly, both programs contain different types of motivation enhancement and cognitive-behavioral strategies, and are at least 8 sessions in length. These programs are consistent with this 9th analysis regarding the components of efficacious teen smoking cessation programming.

The present analysis suggested that programming might best be delivered in a school-based context. However, there are many barriers to delivering health programming in a school context internationally, including lack of understanding of the importance of school health programs, inadequate coordination and lack of a sense of responsibility for student health among school staff, and lack of resources [5]. Key areas to consider include the logistics, cost, time, and human resources required for the implementation of the intervention. A question for consideration is whether the intervention can be implemented on a large scale in a way that will be acceptable by policy makers and the general public, sustainable and cost-effective. Researchers will be able to better examine effect sizes among the theories and modalities, and other variables, when there are a greater number of studies to represent categories, particularly when implemented and evaluated in developing countries.

Future research should make use of appropriate controls, use more standard measures of cessation, and conduct longer follow ups (12 months and perhaps even longer). Future teen smoking cessation studies also might consider use of multiple tobacco use measures (e.g., last 24 hour, last 7 days and last 30 days) to make comparisons consistent across studies. In addition, measures of nicotine dependence (such as the modified FTQ) should be included to assess effectiveness of programming as a function of nicotine dependence [15]. Regular assessment also should include collection of all types of tobacco products used to make sure no substitution effects occur, or that one only quits one type of tobacco use [38]. Also, metrics like cost-effectiveness of treatment per disability/quality of adjusted life years saved should be examined in future teen smoking cessation studies. They may reveal greater cost-effectiveness when compared to adult smoking cessation programs involving persons that are further along on a disease course. Related, types of programming for tobacco users at different levels of use should be further explored

Teen smoking cessation programs are efficacious overall. Also, it is interesting that many of the findings are consistent with those found in the adult cigarette smoking cessation literature, particularly regarding the importance of use of cognitive-behavioral strategies and achieving a sufficient dosage of programming [1]. One difference is that, at the present time, there is little evidence of the efficacy of the use of pharmacologic adjuncts with youth, whereas the evidence appears strong with adults. Future work on the metabolism of pharmacologic adjuncts, youths' patterns of tobacco use, and self-reported withdrawal symptoms might help one to better understand their potential effectiveness among youth.

## Competing interests

The authors declare that they have no competing interests.

## Authors' contributions

SS conceived of the study, did descriptive analysis, and wrote most of the paper. PS carried out the major statistical analyses, and wrote most of the Analysis and Results section for the 64 studies. Both authors read and approved the final manuscript. Please address correspondence to SS.

## Supplementary Material

Additional file 1 Summary data for the 64 studies used in the 2008 updated meta-analysis and their abbreviated references. The information provided are the data used for the updated statistical analysis grouped by parameters examined (theory, modality, number of sessions, length of follow-up), as well as study sample size and design, and an abbreviated references list for the 64 studies. Click here for file
