# Supplementary material for: Youth tobacco use cessation: 2008 update
Source: Tob Induc Dis. 2009 Jan 30;5(1):3. doi: 10.1186/1617-9625-5-3 (PMC2644896; doi:10.1186/1617-9625-5-3)
Supplement: Additional file 1 — Summary data for the 64 studies used in the 2008 updated meta-analysis and their abbreviated references. The information provided are the data used for the updated statistical analysis grouped by parameters examined (theory, modality, number of sessions, length of follow-up), as well as study sample size and design, and an abbreviated references list for the 64 studies. [file 1617-9625-5-3-S1.doc]

Summary data for the 64 studies

| **Study and Country** | **Intervention Theory, Modality, and Number of Sessions/Contacts** | **Design and Total Baseline Sample Size** | **Last Follow-up (months)** | **Absolute Risk Reduction**  **(ARR) and**  **Comments** |
| --- | --- | --- | --- | --- |
| 1. Adelman et al., 2001; US | Cognitive behavioral school-based clinic; #s=8 | Experimental with MPC; n=74 | 3 | 7 |
| 2. Ary et al., 1990; US | Social influences classroom; #s=10 | Experimental with SCC;  n=776 | 12 | 4 |
| 3. Audrey, Holliday, & Campbell, 2006; UK | Cognitive behavioral classroom, #s=about 3 | Experimental with SCC; n=424 teens | 12 | 2; Use of peer nominated  group leaders at teachers |
| 4. Aveyard et al., 1999; UK | Motivation computer-based; #s=6 | Experimental with MPC; n=1090 | 5 | 0; Stages of change |
| 5. Baskerville, Hotte, & Dunkley, 1993; Canada | Motivation system-wide;  #s=2 | Quasi-experimental with SCC; n=331 | 0; 6 months but NR | 17; Contingency-based  Reinforcement |
| 6. Bauman et al., 2000 ; US | Motivation family; #s=5 | Experimental with SCC;  n=110 | 12 | 9 ; Home-based |
| 7. Beaglehole et al., 1978; New Zealand | Social influences classroom; #s=16 | Quasi-experimental with SCC; n=128 | 3 | 0 |
| 8. Bloor et al., 1999; UK | Social influences classroom; #s=about 3 | Quasi-experimental with SCC; n=12 | 3 | -2; Use of peer nominated  group leaders at teachers |
| 9. Brown et al., 2003; US | Motivation medical clinic;  #s=2 | Experimental with MPC; n=191 | 12 | 4 |
| 10. Chan & Witherspoon, 1988; US | Motivation college dormitory; #s=1 | Experimental with MPC; n=40 | 9 | 20; Health risk assessment  plus feedback versus health  risk assessment only |
| 11. Charlton, 1992; UK | Cognitive-behavioral school-based clinic; #s=6 | Quasi-experimental with MPC;  n=87 | 6 | 7 |
| **Study and Country** | **Intervention Theory, Modality, and Number of Sessions/Contacts** | **Design and Total Baseline Sample Size** | **Last Follow-up (months)** | **Absolute Risk Reduction**  **(ARR) and**  **Comments** |
| 12. Cinnomin & Sussman, 1995; US | Cognitive-behavioral school-based clinic; #s=6 | Experimental with OPC, n=60 | 1 | 17; Programs equated for  amount of delivery time |
| 13. Colby et al., 2005; US | Motivation medical clinic; #s=2 | Experimental with MPC; n=85 | 6 | 7 |
| 14. Coleman-Wallace et al., 1999; US | Motivation school-based clinic; #s=8 | Quasi-experimental with SCC; n=351 | 0 | 15; Stages of change |
| 15. Diguisto, 1994; Australia | Cognitive-behavioral school-based clinic; #s=6 | Quasi-experimental with SCC; n=277 | 4 | 7 |
| 16. Dino et al., 2001; US. | Cognitive-behavioral school-based clinic; #s=12 | Quasi-experimental with MPC; n=346 | 5 | 3 |
| 17. Dino, Horn & Meit, 1998; US | Cognitive-behavioral school-based clinic; #s=8 | Quasi-experimental with SCC; n=29 | 2 | 22 |
| 18. Dino et al., 2001; US | Cognitive-behavioral school-based clinic; #s=14 | Quasi-experimental with SCC; n=100 | 5 | 1 |
| 19. Etter, Ronchi, & Perneger, 1999; Switzerland | Other system-wide; #s=2 | Quasi-experimental with SCC; n=582 | 7 | 0; Supply reduction |
| 20. Forster et al., 1998; US | Other system-wide; #s=4 | Experimental with SCC; n=660 | 36 | -5; Supply reduction |
| 21. Glasgow et al., 1999; US | Motivation medical clinic; #s=2 | Experimental with MPC; n=506 | 6 | 4 |

| **Study and Country** | **Intervention Theory, Modality, and Number of Sessions/Contacts** | **Design and Total Baseline Sample Size** | **Last Follow-up (months)** | **Absolute Risk Reduction**  **(ARR) and**  **Comments** |
| --- | --- | --- | --- | --- |
| 22. Greenberg & Deputat, 1978; US | Other school-based clinic; #s=7 | Quasi-experimental with SCC; n=100 | 5 | 8; Affect oriented |
| 23. Hamilton et al., 2005; Australia | Motivation classroom ; #s=8 | Experimental with SCC ; n=2335 | 24 | 4 ; Harm reduction |
| 24. Hancock et al., 2001;  Australia | Social influences system-wide; #s=about 3 | Experimental with SCC ; n=3800 | 42 | 5 |
| 25. Hollis et al., 2005 ; US | Motivation computer-based; #s=3 | Experimental with SCC; n=448 | 24 | 9 ; Stages of change |
| 26. Horn et al., 2004, North Carolina and West Virginia; US | Cognitive-behavioral school-based clinic; #s=12 | Quasi-experimental with MPC; n=250 | 15 | 2 |
| 27. Horn et al., 2005, Florida 1997-98 cohort; US | Cognitive-behavioral school-based clinic; #s=10 | Quasi-experimental with MPC; n=153 | 0 | 17 |
| 28. Horn et al., 2005, Florida 1998-1999 cohort; US | Cognitive-behavioral school-based clinic; #s=10 | Quasi-experimental with MPC; n=305 | 0 | 8 |
| 29. Horn et al., 2005, Florida 1999-2000 cohort; US | Cognitive-behavioral school-based clinic; #s=10 | Quasi-experimental with MPC; n=237 | 0 | 3 |
| 30. Horn et al., 2005, Florida 2000-2001 cohort; US | Cognitive-behavioral school-based clinic; #s=10 | Quasi-experimental with MPC; n=186 | 0 | -1 |
| 31. Horn et al., 2005, North Carolina 2001-2002 cohort; US | Cognitive-behavioral school-based clinic; #s=10 | Quasi-experimental with MPC; n=122 | 0 | 3 |

| **Study and Country** | **Intervention Theory, Modality, and Number of Sessions/Contacts** | **Design and Total Baseline Sample Size** | **Last Follow-up (months)** | **Absolute Risk Reduction**  **(ARR) and**  **Comments** |
| --- | --- | --- | --- | --- |
| 32. Horn et al., 2005, North Carolina and West Virginia 2000-2001; US | Cognitive-behavioral school-based clinic; #s=10 | Quasi-experimental with MPC; n=128 | 0 | 8 |
| 33. Horn et al., 2005; US | Cognitive-behavioral school-based clinic; #s=10 | Quasi-experimental with MPC; n=74 | 3 | 8; American Indians |
| 34. Horn et al., 2007; US | Motivation medical clinic; #s=4 | Experimental with SCC; n=75 | 6 | 0; Motivational  interviewing  in emergency room |
| 35. Horswell & Horton, 1997; Canada | Social influences school-based clinic; #s=3 | Quasi-experimental with SCC; n=36 | 6 | 6 |
| 36. Hotte et al., 1997; Canada | Cognitive behavioral school-based clinic; #s=7 | Quasi-experimental with MPC; n=632 | 6 | 6 |
| 37. Jason, Mollica & Ferrone, 1982; US | Social influences classroom; #s=6 | Quasi-experimental with SCC; n=32 | 17 | 41 |
| 38. Kentala et al., 1999; Finland | Motivation medical clinic; #s=2 | Experimental with SCC; n=148 | 36 | 5; Dental clinic |
| 39. Killen et al., 1988; US | Social influences classroom; #s=20 | Quasi-experimental with SCC; n=180 | 2 | -5 |
| 40. Kohler, Schoenberger, & Phillips, 2005; US | Cognitive-behavioral school-based clinic; #s=14 | Quasi-experimental with SCC; n=492 | 12 | 2 |
| 41. Lando et al., 2003 (and unpublished data); US | Motivation medical clinic; #s=2 | Experimental with MPC; n=344 | 12 | -4 |
| 42. Lazovich et al., 2001; US | Contingency-based court diversion; #s=1 | Experimental with MPC; n=112 | 3 | 0; Attended court diversion  class or paid a fine  (the MPC) |
| **Study and Country** | **Intervention Theory, Modality, and Number of Sessions/Contacts** | **Design and Total Baseline Sample Size** | **Last Follow-up (months)** | **Absolute Risk Reduction**  **(ARR) and**  **Comments** |
| 43. Lipkus et al., 2004, 2006; US | Motivation other public setting; #s=about 2 | Experimental with MPC; n=402 | 8 | 2; Shopping mall and home  telephone counseling |
| 44. Lotecka & McWhinney, 1983; US | Cognitive behavioral school-based clinic; #s=4 | Quasi-experimental with MPC; n=49 | 0 | 0; Coping versus  Information only  (programs equated for  amount of delivery time) |
| 45. Murry, Prokhorov & Harty, 1994; US | Other system-wide; #s=4 | Quasi-experimental with SCC; n=450 | 0 | 2; Supply reduction |
| 46. Myers & Brown, 2005; US | Motivation medical clinic; #s=6 | Quasi-experimental with SCC; n=54 | 6 | 12 |
| 47. Pbert et al., 2006; US | Medical medical clinic ; #s=4 | Experimental with SCC; n=1148 | 3 | 19 ; Nurses as deliverers  of the 5-As quit approach |
| 48. Perry et al., 1980; US | Social influences classroom; #s=4 | Quasi-experimental with SCC; n=243 | 4 | 2 |
| 49. Peterson & Clark, 1986; Australia | Social influences school-based clinic; #s=3 | Quasi-experimental with SCC; n=22 | 1 | 0 |
| 50. Quinlan & McCaul, 2000; US | Motivation school-based clinic; #s=1 | Experimental with SCC; 3 conditions;  n=94 | 1 | 14; Stages of change:  personal match to stage of  change (3%) or  action-oriented stage  (14%), versus SCC (0%) |
| 51. Rigotti et al., 1997; US | Other system-wide; #s=1 | Quasi-experimental with SCC; n=2900 | 24 | 3; Supply reduction |

| **Study and Country** | **Intervention Theory, Modality, and Number of Sessions/Contacts** | **Design and Total Baseline Sample Size** | **Last Follow-up (months)** | **Absolute Risk Reduction**  **(ARR) and**  **Comments** |
| --- | --- | --- | --- | --- |
| 52. Robinson et al., 2003; US | Motivation school-based clinic; #s=4 | Experimental with MPC; n=316 | 12 | -1; For youth caught  smoking; Control was the  CDC “I Quit” self-help  Guide |
| 53. Rodgers et al., 2005; New Zealand | Cognitive-behavioral computer-based; #s about 3 | Experimental with SCC; n=617 | 6 | 2; Use of cell phone text  Messaging |
| 54. Stoddard et al., 2005; US | Social influences other public setting; #s=8 | Experimental with SCC; n=560 | 12 | 7; Work-sites |
| 55. Suedfeld et al., 1972; US | Other sensory deprivation; #s=1 | Experimental with SCC;  n=40 | 3 | 0; Affect-oriented |
| 56. Sussman, Burton et al., 1995; US | Cognitive behavioral school-based clinic; #s=5 | Experimental with SCC;  n=244 | 3 | 7 |
| 57. Sussman, Dent & Lichtman, 2001; US | Motivation school-based clinic; #s=8 | Experimental with SCC;  n=335 | 5 | 9 |
| 58. Sussman, Dent & Stacy, 2002; US | Motivation school-based classroom drug abuse prevention program with 1 tobacco cessation session; #s=12 | Experimental with SCC;  n=583 | 12 | 5 |
| 59. Sussman et al., 2007; US | Motivation classroom; #s=8 | Experimental with SCC;  n=461 | 12 | 4 |
| 60. Winkleby et al., 2004; US | Social influences classroom; #s=5 | Experimental with MPC; n=813 | 6 | 5; Tobacco-focused advocacy  Intervention versus  modified drug abuse  prevention program;  Programs equated for  amount of delivery time |
| 61. Yiming et. al., 2000; Singapore | Medical model medical clinic; #s=12 | Experimental with SCC; n=330 | 3 | -1; Lazar versus sham  Acupuncture |
| **Study and Country** | **Intervention Theory, Modality, and Number of Sessions/Contacts** | **Design and Total Baseline Sample Size** | **Last Follow-up (months)** | **Absolute Risk Reduction**  **(ARR) and**  **Comments** |
| 62. Zack et al., 2005; US | Cognitive-behavioral school-based clinic; #s=6 | Experimental with SCC; n=125 | 12 | 8 |
| 63. Zavela, Harrison & Owens, 1991; US | Medical model school-based clinic; #s=5 | Experimental with MPC ; n=42 | 1 | 11 |
| 64. Zheng et al., 2004; China | Motivation school-based clinic; #s=8 | Single group multiple baseline, within group control; n=46 | 4 | 0-8%;  4-11%; in the 2006 review  the immediate posttest  results were used and have  not been replaced in the  current paper with the 4  month follow-up results |

Notes. SCC=standard care control; MPC=minimal program control

Abbreviated references for the 64 studies

| 1. W. Adelman et al., *Pediatrics*, 2001, 107: E50 (11 pages). |
| --- |
| 2. D. Ary et al., *Journal of Behavioral Medicine*, 1990, 13, 281-296. |
| 3. S. Audrey, Holliday, & Campbell, *Social Science & Medicine*, 2006, 63, 320-334. |
| 4. P. Aveyard et al., *British Medical Journal*, 1999, 319, 948-953. |
| 5. B. Baskerville, Hotte, & Dunkley, *Community Health Research Unit report*, 1993, University of Ottawa. |
| 6. K. Bauman et al., *Prevention Science*, 2000, 1, 227-237. |
| 7. R. Beaglehole et al., *New Zealand Medical Journal*, 1978, 87, 278-280. |
| 8. M. Bloor et al., *Health Education Journal*, 1999, 58, 17-25. |
| 9. R. Brown et al., *Tobacco Control*, 2003, 12, iv3-iv10. |
| 10. C.W. Chan & Witherspoon, *Journal of General Internal Medicine*, 1988, 3, 555-559. |
| 11. A. Charlton, *Health Education Research*, 1992, 7, 249-257. |
| 12. D. Cinnomin & Sussman, *unpublished study*, summarized in S. Sussman et al., 1995, *Developing school based tobacco use prevention and cessation programs*. Newbury Park: Sage. |
| 13. S. Colby et al., *Addictive Behaviors*, 2005, 30, 865-874. |
| 14. D. Coleman-Wallace et al., *The Journal of School Health*, 1999, 69, 314-319. |
| 15. E. Diguisto, Chapter 6, *Interventions for Smokers* (R. Richmond, Ed.),1994, Williams & Wilkins. |
| 16. G. Dino et al., *The Journal of School Nursing*, 2001, 17, 90-97. |
| 17. G. Dino, Horn & Meit, *Health Education*, 1998, 6, 230-241. |
| 18. G. Dino et al., *Preventive Medicine*, 2001, 33, 600-605. |
| 19. J.-F. Etter, Ronchi, & Perneger, *Journal of Epidemiology and Community Health*, 1999, 53, 710-715. |
| 20. J. Forster et al., *American Journal of Public Health*, 1998, 88, 1193-1198 |
| 21. R. Glasgow et al., *Journal of Consulting and Clinical Psychology*, 1999, 67, 1009-1011. |
| 22. J. Greenberg & Deputat, *The Journal of School Health*, 1978, 48, 498-502. |
| 23. G. Hamilton et al., *Addiction*, 2005, 100, 689-700. |
| 24. L. Hancock et al., *Preventive Medicine*, 2001, 32, 332-340. |
| 25. J. Hollis et al., *Pediatrics*, 2005, 115, 981-989. |
| 26. K. Horn et al., *Journal of Adolescent Research*, 2005, 20, 640-661, Florida 1997-98 cohort. |
| 27. K. Horn et al., *Journal of Adolescent Research*, 2005, 20, 640-661, Florida 1998-99 cohort. |
| 28. K. Horn et al., *Journal of Adolescent Research*, 2005, 20, 640-661, Florida 1999-2000 cohort. |
| 29. K. Horn et al., *Journal of Adolescent Research*, 2005, 20, 640-661, Florida 2001-2002 cohort. |
| 30. K. Horn et al., *Journal of Adolescent Research*, 2005, 20, 640-661, West Virginia 2000-2001 cohort. |
| 31. K. Horn et al., *Journal of Adolescent Research*, 2005, 20, 640-661, North Carolina 2001-2002 cohort. |
| 32. K. Horn et al., *Health Education*, 1999, 5, 192-206. |
| 33. K. Horn et al., *Preventing Chronic Disease: Public Health Research, Practice, and Policy*, 2005, 2, 1-11. |
| 34. Horn et al., *Preventing Chronic Disease: Public Health Research, Practice, and Policy*, 2007, 4, 1-12. |
| 35. L. Horswell & Horton, *Community Action Initiatives Program report*, 1997, Health *Canada*, Ottawa. |
| 36. A. Hotte et al., *Community Action Initiatives Program report*, 1997, Health *Canada*, Ottawa. |
| 37. L. Jason, Mollica & Ferrone, *Preventive Medicine*, 1982, 11, 96-102. |
| 38. J. Kentala et al., *Preventive Medicine*, 1999, 29, 107-111. |
| 39. J. Killen et al., *Journal of the American Medical Association*, 1988, 260, 1728-1733. |
| 40. C. Kohler, Schoenberger, & Phillips, Paper presentation at *Society for Research on Nicotine and Tobacco (SRNT) 2005 Annual Meeting*, 2005, Prague, Czech Republic. |
| 41. H Lando et al., *Youth Tobacco Research Meeting and Tobacco Synthesis Meeting*, 2003 (and unpublished data). |
| 42. D. Lazovich et al., *American Journal of Public Health*, 2001, 91, 1790-1791 (& Lazovich Master’s Thesis) |
| 43. I.M. Lipkus et al., *Health Psychology*, 2004, 2006, 23, 397-406. |
| 44. L. Lotecka & McWhinney, *International Journal of the Addictions*, 1983, 18, 479-490. |
| 45. D. Murry, Prokhorov & Harty, *Preventive Medicine*, 1994, 23, 54-60. |
| 46. M. Myers & Brown, *Psychology of Addictive Behaviors*, 2005, 19, 230-233. |
| 47. L. Pbert et al., *Preventive Medicine*, 2006, 43, 312-320. |
| 48. C. Perry et al., *American Journal of Public Health*, 1980, 70, 722-725. |
| 49. A. Peterson & Clark, *Psychological Reports*, 1986, 58, 179-185. |
| 50. K. Quinlan & McCaul, *Health Psychology*, 2000, 19, 165-171. |
| 51. N. Rigotti et al., *The New England Journal of Medicine*, 1997, 337, 1044-1051. |
| 52. L.A. Robinson et al., *Tobacco Control*, 2003, 12 (Suppl. IV), iv26-iv33. |
| 53. Rodgers et al., *Tobacco Control*, 2005, 14, 255-261. |
| 54. A.M. Stoddard et al., *Cancer Causes and Control*, 2005, 16, 1159-1164. |
| 55. P. Suedfeld et al., *International Journal of the Addictions*, 1972, 7, 721-733. |
| 56. S. Sussman, Burton et al., Book: *Developing School-based Tobacco Use Prevention and Cessation Programs*, 1995, Sage |
| 57. S. Sussman, Dent & Lichtman, *Addictive Behaviors*, 2001, 26, 425-438. |
| 58. S. Sussman, Dent & Stacy, *American Journal of Health Behavior*, 2002, 26, 354-365. |
| 59. S. Sussman et al., *Addictive Behaviors*, 2007, 32, 3005-3014. |
| 60. M. Winkleby et al., *Archives of Pediatric and Adolescent Medicine*, 2004, 158, 269-275. |
| 61. C. Yiming et. al., *American Journal of Chinese Medicine*, 2000, 28, 443-449. |
| 62. S.L. Zack et al., Paper presentation at *2005 National Conference on Tobacco or Health*, 2005, Chicago, Illinois. |
| 63. K. Zavela, Harrison & Owens, *American Public Health Association 119^th^ Meeting Poster Presentation*, 1991. |
| 64. H. Zheng et al., *Addictive Behaviors*, 2004, 29, 1725-1733. |
